# Patterning order and disorder with an angle: modeling single-layer dual-phase nematic elastomer ribbons†

**DOI:** 10.1039/c8ra09375j

**Published:** 2019-03-18

**Authors:** Vianney Gimenez-Pinto, Fangfu Ye

**Affiliations:** Beijing National Laboratory for Condensed Matter Physics, CAS Key Laboratory of Soft Matter Physics, Institute of Physics, Chinese Academy of Sciences Beijing 100190 China vgimenezpinto@temple.edu; Department of Chemical Engineering, Columbia University USA; School of Physical Sciences, University of Chinese Academy of Sciences Beijing 100049 China; Songshan-Lake Materials Laboratory Dongguan 523808 China

## Abstract

Liquid crystal polymer networks have demonstrated a rich variety of actuation behavior and stimulus-responsive properties. Actuation in these single-phase materials is given by spatial variations in their liquid crystal director microstructure in balanced coexistence with rubber elasticity. However, experimental studies have shown that complex actuation in elastomeric materials can also be engineered by combining well-defined isotropic regions along with liquid crystalline ordered regions. *Via* finite element elastodynamics simulations, we investigate the actuation behavior of these dual-phase nematic elastomer ribbons based on several key design factors: director orientation, pattern orientation, as well as domain and sample size. We demonstrate the variety of shapes that these materials can exhibit, including twisting, bending, accordion folding, and hybrid flat-helix states. Overall, our simulations show an exceptional agreement with experimental observations, providing light for the further development of soft stimulus-responsive materials with complex microstructures.

## Abstract 2

We study the rich actuation variety displayed by elastomers that combine well-defined isotropic regions and liquid crystalline regions. Design factors for actuation include orientation of director and pattern, domain-size and sample-size.

## Graphical abstract

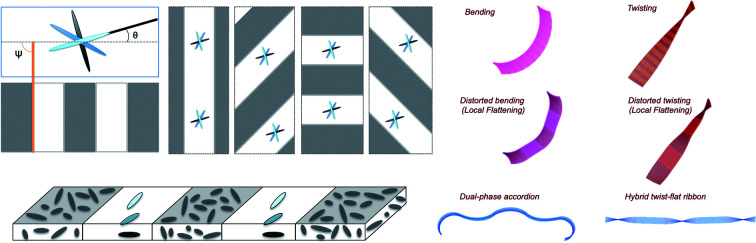

Liquid crystal polymer networks are hybrid materials that encode complex shape transformation and stimulus-responsive capabilities given the coexistence of liquid crystalline order and a crosslinked polymer network with characteristic rubber-like elasticity. By engineering complex microstructures in the nematic director field, complex shape actuation under external stimulus can be custom-designed in these materials. A wide variety of nematic director patterns in delicate combination with the elastic response, have demonstrated an exquisite repertoire of shapes, including the formation of cones,^1^ pyramids where Gaussian curvature is suppressed by in-plane variations of the director,^2^ chiral helicoids and elastic springs,^3–8^ foldable actuators,^6,9–13^ and other complex bio-inspired morphologies.^14,15^ The vast majority of these single-phase designs are based on spatial variations of the nematic director field within the sample. When external stimuli such as light, temperature change, variation in chemical environment, *etc.* change the liquid crystalline order parameter, inner strains arise in the material and produce a macroscopic shape deformation. A non-uniform director microstructure produces a non-uniform distribution of strains under external stimulus, driving then complex actuation.

Nonetheless, additional design approaches can be implemented towards engineering complex spatial distribution of inner strains in the material and, as a result, complex actuation under external-stimuli. Recently, Liu *et al.*^16^ synthetized *single-layer dual-phase* thin-film elastomer actuators, which ingeniously combine well-defined isotropic regions and twist-nematic liquid crystalline regions. Yang and Zhao^17^ also achieved synthetizing single-layer hybrid elastomer samples within their studies implementing non-uniform optical inscription of isotropic–liquid crystalline patterns. Furthermore, Katsonis and coworkers^18^ recently developed elastomer films with stripes of isotropic and liquid crystal domains and implemented them for the study of high-power light-driven actuation in paired chiral ribbons. Similarly to single-phase liquid crystal elastomers, these dual-phase materials can display a variety of out of plane actuation behavior covering chiral twisting and bending, as well as the formation of accordion folds. Here, actuation is encoded within the nematic director microstructure coexisting in combination with the over-imposed in-plane isotropic pattern.

Combining order and disorder in the microstructure of liquid crystal elastomers has also been implemented in *bi-layer dual-phase* materials within the past 15 years. Ikeda and coworkers^19^ first introduced photo-responsive dual-phase bilayer elastomers with one isotropic layer and an anisotropic smectic layer. Also, Dai *et al.*^20^ synthetized humidity-responsive dual-layer elastomers with an isotropic layer in coexistence with a nematic layer with splay configuration. In both cases, the stacked arrangement of one isotropic and one liquid crystalline layer added mechanical strength to the sample, avoiding fracture and cracking. We note that these mechanically reinforced *bi-layer dual-phase* strips arrange order and disorder *along sample thickness*. Conversely, *single-layer dual-phase* materials implement *in-plane* order–disorder patterns, allowing further options for design parameters towards engineering complex actuation, such as significant variations in the size of isotropic regions as well as the use of in-plane angles in the material microstructure.

In this work, we perform finite-element elastodynamics simulations for investigating the actuation of these novel single-layer dual-phase materials given their order–disorder patterned microstructure as well as director configuration in the nematic regions. This numerical method had successfully demonstrated a wide variety of in-plane and out-of-plane actuation behavior in single-phase nematic elastomers including chiral bending,^8^ twisting,^8,22^ cone and anti-cone (saddle) formation,^21^ accordion folds,^9^ origami boxes,^11^ and bas-relief designs,^21^ given by different complex director microstructures. Also, finite element elastodynamics has been implemented for studying the actuation of smectic elastomers.^22^ However, until now there is no record of this approach being implemented for the study of dual-phase patterned elastic solids. More importantly, given the freshness of single-layer experimental realizations, the current literature still lacks theoretical and numerical studies for these novel dual-phase rubbery materials.

Here, we focus on single-layer dual-phase elastomers with a 90 degree twist-nematic configuration in the nematic region, analyze the effect of a varying offset angle in director macrostructure as well as a varying offset angle in the orientation of the isotropic pattern. Overall, we demonstrate the different cases and geometries where curling, folding, bending and accordion-like folds appear under external stimulus in these materials. Our study shows that dual phase elastomer ribbons can exhibit similar actuation to their single-phase counterparts, or a modified deformation by the influence of inactive isotropic domains, depending on their size relative to sample size.

In materials with a striped isotropic–nematic pattern, narrow inactive isotropic stripes do not distort overall morphology after actuation, and only changes in the magnitude of the actuation and stimulus response are observed. Wider isotropic stripes can distort and/or isolate the effect of their adjacent active nematic regions affecting overall out-of-plane actuation. In these later cases, we observe flat inactive regions in coexistence with active morphing regions. Wide-stripe samples exhibit distorted-bending, distorted-twisting, accordion folds and hybrid twist-flat morphologies depending on stripe width and angular offset. Our simulation study sheds light on the different morphologies reported by experimental groups that previously synthetized these materials, and give insights on the key design factors for engineering actuation in smart materials by patterning order and disorder with an angle.

*Methods*: We implement finite element elastodynamics simulations as developed by Selinger and coworkers.^23^ For this, each three-dimensional sample is discretized to an unstructured tetrahedral mesh with an open source CAD software.^24^ The system is described *via* the Hamiltonian
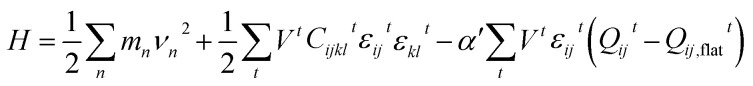
where implementing the Green-Lagrange non-linear strain tensor *ε*_*ij*_ = ∂_*i*_*u*_*j*_ + ∂_*j*_*u*_*i*_ + ∂_*i*_*u*_*k*_∂_*j*_*u*_*k*_ allows large deformations. *C*_*ijkl*_ corresponds with the elastic stiffness tensor of an isotropic elastic solid (rubber). Coupling between nematic order, *Q*_*ij*_ = *S*〈*n*_*i*_*n*_*j*_ − *δ*/3〉 and strain *ε*_*ij*_ is given by the term *α*(*Q*_*ij*_ − *Q*^ref^_*ij*_)*ε*_*ij*_. Here, nematic order is represented as a piece-wise function within tetrahedral elements *t*, as well as the strain tensor. Deformation in each tetrahedron is calculated *via* linear mapping functions on the displacement vectors of tetrahedra nodes *n*.§§Here the index *n* enumerates the nodes in the mesh, and the symbol *n*_*i*_ represents the nematic director. Nodes move *via F* = *ma*, where *F* is calculated as a derivative of the free energy with respect to node position. Node mass follows the lump-mass approximation. Particle trajectories are calculated forwards in time *via* the velocity-Verlet algorithm. A dissipative force proportional to node momentum is implemented to allow the system to reach equilibrium. Further details on this finite element elastodynamics algorithm can be found in previous work.^11,21,23,25^

For modeling dual phase actuators, we use the imprinted director field configuration to describe the spatial distribution of nematic order and disorder. Director in the nematic regions has a 90 degree twist between top and bottom substrates. An additional offset angle *θ* is included in the configuration, where *θ* describes the angle between the director in the bottom substrate and the sample long axis. Isotropic regions were introduced by a random three-dimensional orientation of the nematic director *n*_*i*_: sample is treated as a random poly-domain elastomer with domain size matching unstructured tetrahedron size.

When modeling these materials *via* finite-element studies, we discretize and define the nematic director and order parameter within each tetrahedral element and ensure that the number of tetrahedra is above the minimum requirements for a smooth variation of the nematic director in the nematic regions. We note that isotropic state in liquid crystal materials and liquid crystalline polymers is well described by a null scalar order parameter *S*. However, nematic order (or lack of it) is described by two quantities: the nematic order parameter *S* and the nematic director describing spatial average molecular orientation. Depending on domain size, poly-domain materials can be isotropic at a macroscopic level and still present orientational order at smaller scales. For computational simplicity, we set the sample to have a well-defined but random director and non-zero *S* within each unstructured tetrahedral element. Then, the lack of structure (isotropy) in the fine sample discretization in combination with random orientation of the director inside each tetrahedron becomes sufficient to model the overall disordered configuration of the isotropic regions. However, we note that the validity of this approximation depends on mesh type (unstructured tetrahedral) as well as grade of discretization.

The isotropic–nematic pattern over-imposed in the sample follows alternate stripes with uniform size. Stripes also present a characteristic angle *ψ* between the stripe border and the sample long axis, see Fig. 1. This simulation setup was designed to replicate the fabrication technique for dual-phase elastomer ribbons described by Liu *et al.*^16^ Magnitude of external stimulus is encoded in the parameter *αδS*, in consistency with previous numerical studies implementing finite element elastodynamics in liquid crystal elastomers.^8,9,11,21^ External stimulus is applied during a transient time, and then sample is allowed to respond and find equilibrium. In this work, we use two samples with different lengths: sample 1 has aspect ratio 50-10-1, 13 213 nodes, 61 953 tetrahedral elements; sample 2 has aspect ratio 200-10-1, 65 924 nodes, and 291 711 tetrahedra. To ensure a smooth variation of the director along sample thickness and adequate convergence of the finite element elastodynamics code, fine spatial and time discretization is necessary. Thus, mesh requires at least five elements along its thickness in addition to a simulation time-step d*t* = 1 × 10^−5^ au. We obtained these criteria by monitoring the volume of tetrahedral elements. Obtaining elements with negative volume during the simulation is an indicator of non-convergence of the finite element method, producing non-physical results.

**Fig. 1:**
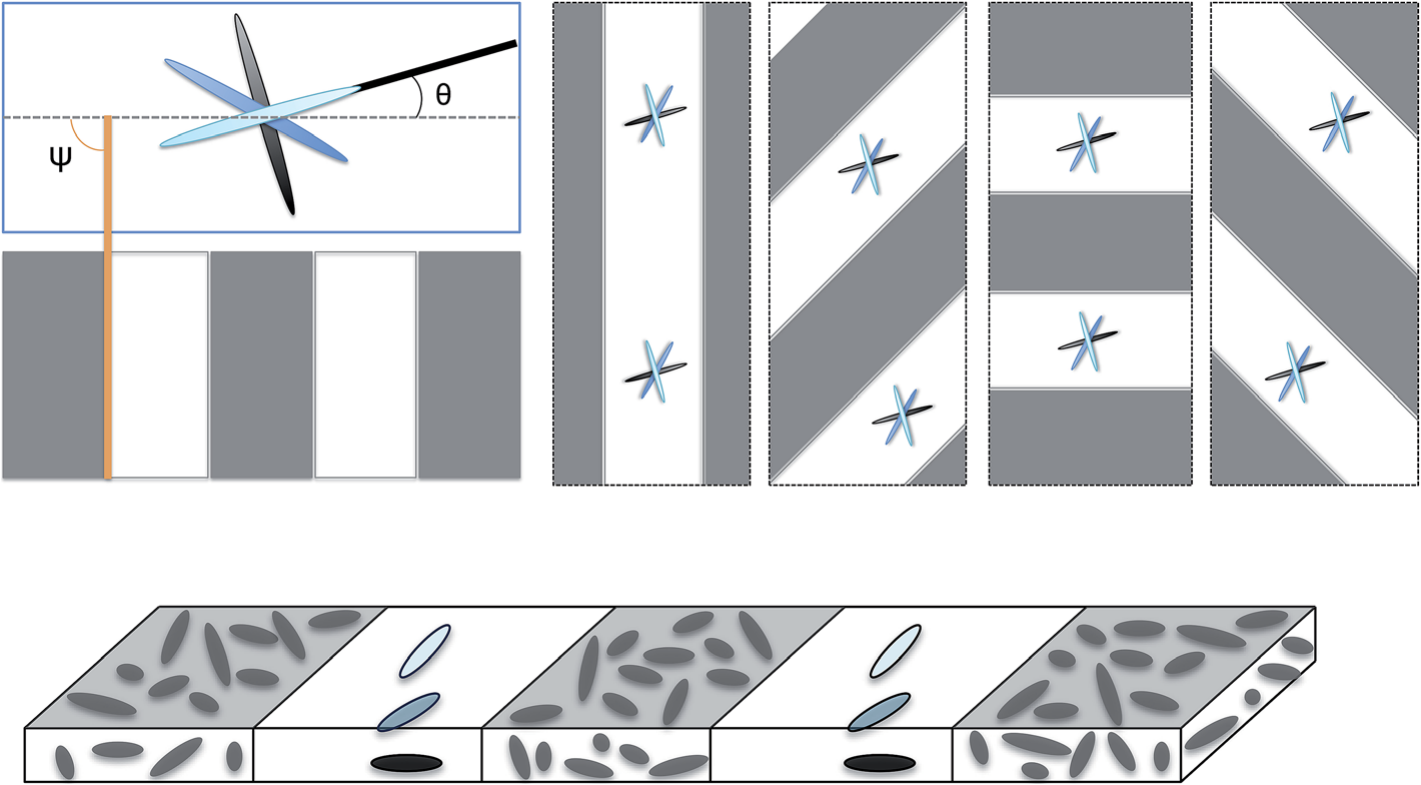
Schematics of dual-phase nematic actuators: white regions have nematic order with 90 degree twist between top and bottom substrates. Grey regions correspond with isotropic states. In the fabrication of these materials,^16^ disordered regions can be patterned parallel to, perpendicular to or at a specific angle *ψ* with the long axis of the ribbon. Thus, materials have two characteristic orientations: an angular offset *θ* for the 90 degree twist and the angular parameter for the mask *ψ*.

*Undistorted and distorted chiral bending and twisting*: Fig. 2 shows the side view of dual-phase elastomers with stripe width/thickness = 2.0, for different values of *angular offset in director microstructure*, *θ* = 0, 45 and 90 degrees and *in the isotropic stripes orientation*, *ψ* = 0, 45, 90 and 135 degrees. In this case, the width of the isotropic stripes is not significant for a local effect in the sample morphology after actuation. We find no distortion of the twisting and bending preset by the twist-nematic director and angular offset *θ*. Samples show overall bending and twisting similar to single-phase nematic samples, with changes observed in the magnitude of mean and Gaussian curvature as well as on the helical pitch. This narrow-stripe case agrees with the actuation behavior observed in experiments by Katsonis and coworkers,^18^ in which the presence of isotropic stripes alternating with twist-nematic stripes does not produce a macroscopic distortion of the helical twist morphology after illumination. We found, however, visible changes in the sample thickness occur according to the stripe patterned design. Fig. 3a shows the enlarged *xz* (width-thickness) cross-section of ribbon with *θ* = 0, *ψ* = 0. Given the increase in nematic order, active nematic regions become thinner than inactive isotropic regions. Fig. 3b shows a surface-detailed image of ribbon *θ* = 0, *ψ* = 90, exhibiting undulations on the surface as well as along sample long axis. Then, the effect of this narrow-stripe order–disorder microstructure becomes apparent by distorting these samples at their smallest dimension. We note that edge-distortions were also observed in experimental studies by Katsonis and coworkers.^18^

**Fig. 2:**
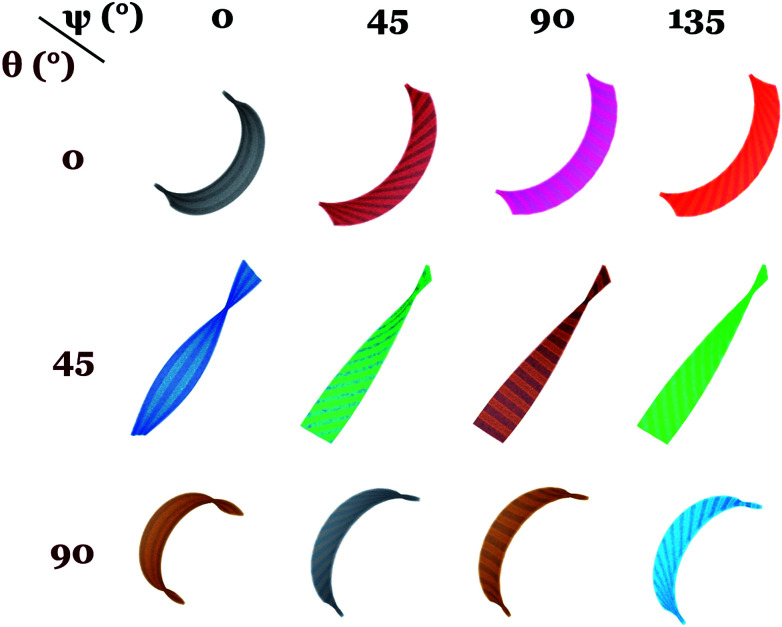
Dual phase elastomers with narrow stripes, stripe width/thickness = 2.0, and sample aspect ratio 50 : 10 : 1. Stimulus response is *αδS* = +1.71. Narrow stripes with isotropic order do not affect the overall shape of the actuator. Chirality changes as a function of angular offset *θ* in nematic director and remains un-affected by changes in angle *ψ* for the stripe pattern orientation.

**Fig. 3:**
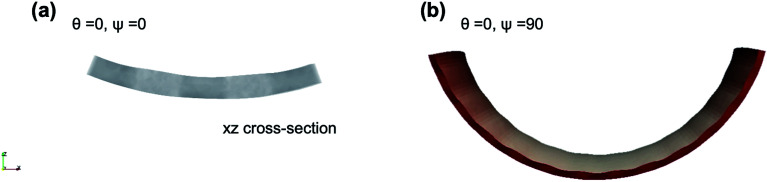
Actuated thickness in dual-phase ribbons with narrow stripes: stripe width/thickness = 2.0, and sample aspect ratio 50 : 10 : 1. Stimulus response is *αδS* = +1.71. (a) Cross-section (plane *xz*) of ribbon *θ* = 0, *ψ* = 0 shows isotropic regions (lighter grey) visibly thicker than active nematic regions. (b) Ribbon *θ* = 0, *ψ* = 90 shows undulations in both surface and edges – color represent the *z*-component of the surface normal.

*Undistorted chiral bending* appears in samples with *θ* = 0 and 90 degrees. We find that these samples have opposite chirality to the other, a result that agrees with previous work on single-phase off-axis twist-nematic elastomers,^8^ as well as studies that characterize the oriented nature of chirality.^26^*Undistorted chiral helicoids* with negative Gaussian curvature and straight sample midline are found for *θ* = 45 degrees. These show significant variations in the helical pitch between samples with vertical stripes (*ψ* = 0) and the rest of the simulation set. This difference is due to the reduced area covered by the isotropic domains in this specific case. We found no change in sample chirality when changing the orientation of the isotropic lines. While chirality has a well-known and well-defined dependence on orientation in anisotropic materials, our simulations show that creating an orientation by patterning isotropic regions with a *ψ* angle does not produce a similar behavior in the overall sample shape. The effect of *θ* orientation in the anisotropic liquid crystalline order was accurately found in this study.

Fig. 4 shows the equilibrium shapes obtained by finite element elastodynamics in a sample with stripe width/thickness = 10.0. In this simulation set, we find *local flattening of the ribbon given by the wider inactive isotropic regions*. A case that agrees with the observations of local flattening in a variety of striped ribbon samples by Liu *et al.*^16^ Yang and Zhao^17^ also observed a local flattening in the actuation of their single-layer dual-phase sample patterned with a diamond-shaped liquid crystalline region. For *ψ* = 45, 90 and 135 degrees, the inactive isotropic regions produce a *distortion of macroscopic bending* (samples with *θ* = 0 or 90 degrees) *and twisting* (samples with *θ* = 45 degrees) in the ribbons, with most of the effect occurring along the edge-line between the isotropic and nematic stripes. Given the non-uniform director and non-uniform strains within the sample, this edge-line between adjacent stripes localizes high strains producing a more pronounced distortion in this region.

**Fig. 4:**
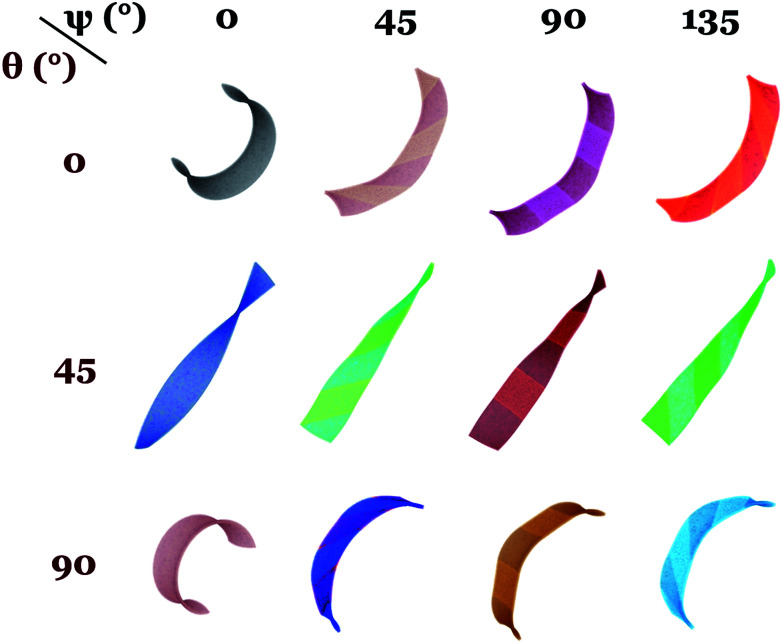
Dual phase elastomers with wide stripes, stripe width/thickness = 10.0, sample aspect ratio 50 : 10 : 1, and *αδS* = +1.71. As the size of the isotropic region increases, its flattening effect becomes visible in overall morphology after actuation. Distortion is clearer along the edge-line of the isotropic–nematic regions and changes in *ψ* affect the local Gaussian curvature of the shape. We find particular interest in the cases with *ψ* = 45 and 90 degrees, while oblique stripe patterning does not show significant effect in helicoid and chiral-bend actuation, horizontal stripes visibly change overall helicoid morphology to an alternating saddle-flat shape.

Again, we find that changing the orientation of the disordered stripes does not affect ribbon's macroscopic chirality. Undistorted cases are found for *ψ* = 0, where a single nematic stripe fully covers the full sample area, corresponding with a single-phase elastomer sample with off-axis angle *θ*.

*Accordion-like folds and hybrid twist-flat ribbon*: While the results in the previous section show the effect of inactive isotropic domains in ribbon actuation, none of the simulated samples (Fig. 4) with alternating isotropic–nematic stripe configuration exhibits an accordion-like morphology similar to the alternating twist-nematic stripe pattern developed by Broer and coworkers.^9^ Sample with *θ* = 0 and *ψ* = 90 (Fig. 4) has an overall C-shape after actuation instead of accordion-like. This is due to small stripe width values limited by the given ribbon length of samples with aspect ratio 50 : 10 : 1. In order to obtain accordion-like folds in dual-phase elastomers, even wider isotropic stripes are needed to isolate local bending occurring in the nematic domains. Simulations show that accordion-like folds are obtained in a sample with aspect ratio 200-10-1, 65 924 nodes, 291 711 elements and stripe width-to-thickness ratio = 40.0. As expected, sample with *θ* = 0 and *ψ* = 90, forms well-controlled accordion folds and changing *θ* to 90 degrees inverts the out-of-plane direction of the folds.

For *θ* = 45, we obtained a hybrid-twist actuator where nematic regions form helicoids with negative Gaussian curvature and isotropic regions remain flat and inactive. Even though the width of isotropic domains is significant in this sample, we find the same actuation behavior when changing stripe angle *ψ*: dual-phase accordion, hybrid-helix and backwards accordion for *θ* = 0, 45 and 90 degrees respectively (see Fig. 5). This is due to the use of a narrow sample. Geometrically, such a narrow sample suppresses the effect from changing the stripe pattern orientation. Increasing the width of samples could model a richer behavior where overall bending (similar to Fig. 2 and 4) can be observed.

**Fig. 5:**
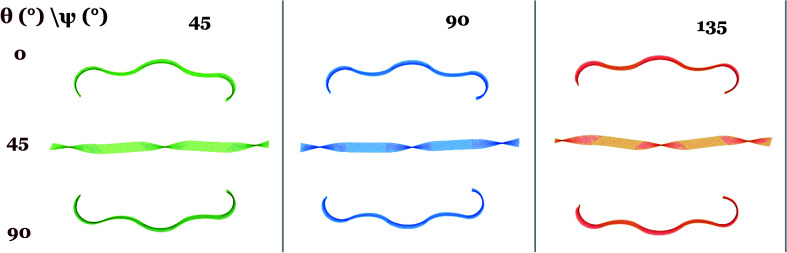
Dual-phase accordion and dual-phase twist ribbon given by changes in director angular offset. From top to bottom: accordion folds, hybrid-twist, and backwards accordion. *αδS* = +1.71. Isotropic stripes have *ψ* = 45, 90 and 135 degrees angle with the ribbon long axis. Given the narrow ribbon width used in the simulation, changes in *ψ* do not affect the overall shape.

The time evolution of the hybrid twist-flat actuation also presents an interesting behavior (see ESI Video 1†). Immediately after the external stimulus, the initial morphing of the material corresponds with an arrangement of helicoids with: active regions having macroscopic chirality given by the handedness of its twist-nematic microstructure, and isotropic regions showing transient twisting with opposite chirality. This deformation of the isotropic regions is given by the adjacent strains produced by the deformation of active nematic regions. At subsequent time steps, isotropic regions dissipate adjacent strains and system reaches a state with twist-flat-twist overall morphology. This relaxation of adjacent strains and flattening in the isotropic regions occurs within 240 000 time steps, while the complete system reaches a final hybrid-flat-twist state in 2 600 000 time steps. We note that initial work by Liu *et al.*^16^ on single-phase dual elastomers reports continuous oscillations in the sample actuation. These could be given by an interplay between strain relaxation by the isotropic domains in combination with a time-varying non-uniform external-stimulus given by laboratory settings. However, the current study is focused on capturing the overall deformation of these materials applying a uniform stimulus rather than further analyzing causes for continuous motion.

We note that the main ribbon morphologies observed in single-layer dual phase ribbons: twisting and chiral bending, are programed by the twist-nematic microstructure along the sample thickness in the active nematic regions. In these materials, the role of the isotropic–nematic pattern is to create in-plane variations of this ordered microstructure. Given isotropic region size, isolation of active nematic regions can occur, affecting overall shape morphology. However, the implementation of patterned isotropic–nematic domains is not strictly limited to twist-nematic configurations. With set anchoring conditions, nematic regions can have other director configurations like splay, radial and azimuthal disclinations with defined topological charge, or more complex custom-made three-dimensional director fields. Furthermore, the use of a crosslinking gradient along sample thickness creates an additional source of anisotropy useful as a design parameter for the director configuration of the nematic regions.^4^ Extending dual-phase elastomers patterns to more complex director configurations would allow engineering a wide variety of morphologies still to be studied.

Overall, we numerically modeled the stimulus-response shape deformation of samples with alternating isotropic–nematic stripes. The clever design of these dual-phase twist-nematic elastomers allows engineering actuators with twist, chiral bending and accordion-like folds. We demonstrated the size-effect of the isotropic stripes in determining the overall shape of the material. In the case of narrow stripes (2.0 stripe width/thickness), shape is practically unaffected by the isotropic regions; while for wider stripes (40.0 stripe width/thickness) isotropic domains can isolate local actuation in the nematic region and create a modified morphology different from the single-phase counterpart. This study from a numerical perspective gives an insight on the shape-morphing capabilities and actuator design features of dual-phase nematic elastomers, and thus opens the door to a distinct approach for engineering complex actuation in liquid crystal polymeric films.

## Conflicts of interest

Authors declare no conflict of interest.

## Supplementary Material

RA-009-C8RA09375J-s001

RA-009-C8RA09375J-s002
