# Supplementary material for: Patterning order and disorder with an angle: modeling single-layer dual-phase nematic elastomer ribbons
Source: RSC Adv. 2019 Mar 18;9(16):8994–9000. doi: 10.1039/c8ra09375j (PMC9062075; doi:10.1039/c8ra09375j)
Supplement: RA-009-C8RA09375J-s002 [file RA-009-C8RA09375J-s002.pdf]

Electronic Supplementary Material (ESI) for RSC Advances.
This journal is © The Royal Society of Chemistry 2019

Supplementary Information for

Patterning order and disorder with an angle: Modeling single-layer dual-phase nematic elastomer ribbons

Vianney Gimenez-Pinto [1,2||] and Fangfu Ye [1,3,4]*

[1]*Beijing National Laboratory for Condensed Matter Physics & CAS Key Laboratory of Soft Matter Physics, Institute of Physics, Chinese Academy of Sciences, Beijing 100190, China*
[2]*Department of Chemical Engineering, Columbia University*
[3]*School of Physical Sciences, University of Chinese Academy of Sciences, Beijing 100049, China*
[4]*Songshan-Lake Materials Laboratory, Dongguan 523808, China*

**Current Addresses: Department of Physics and Astronomy, University of Pennsylvania and Department of Physics, Temple University*

[||]*Corresponding author: vgimenezpinto@temple.edu*

Supplementary Video 1*: Actuation of a hybrid flat-twist ribbon with stripe-width/thickness ratio=40.0. Stripe pattern has an angle ψ =90 degrees and the twist-nematic director configuration has an offset angle θ =45 degrees. During a transient time, sample show chiral saddles in active nematic region and saddles with opposite chirality in the inactive isotropic regions. This short-lived deformation (first second of video) is consequence of adjacent strains produced by the morphing of the nematic stripes. Isotropic regions are then allowed to relax and find equilibrium in a flat state.*
